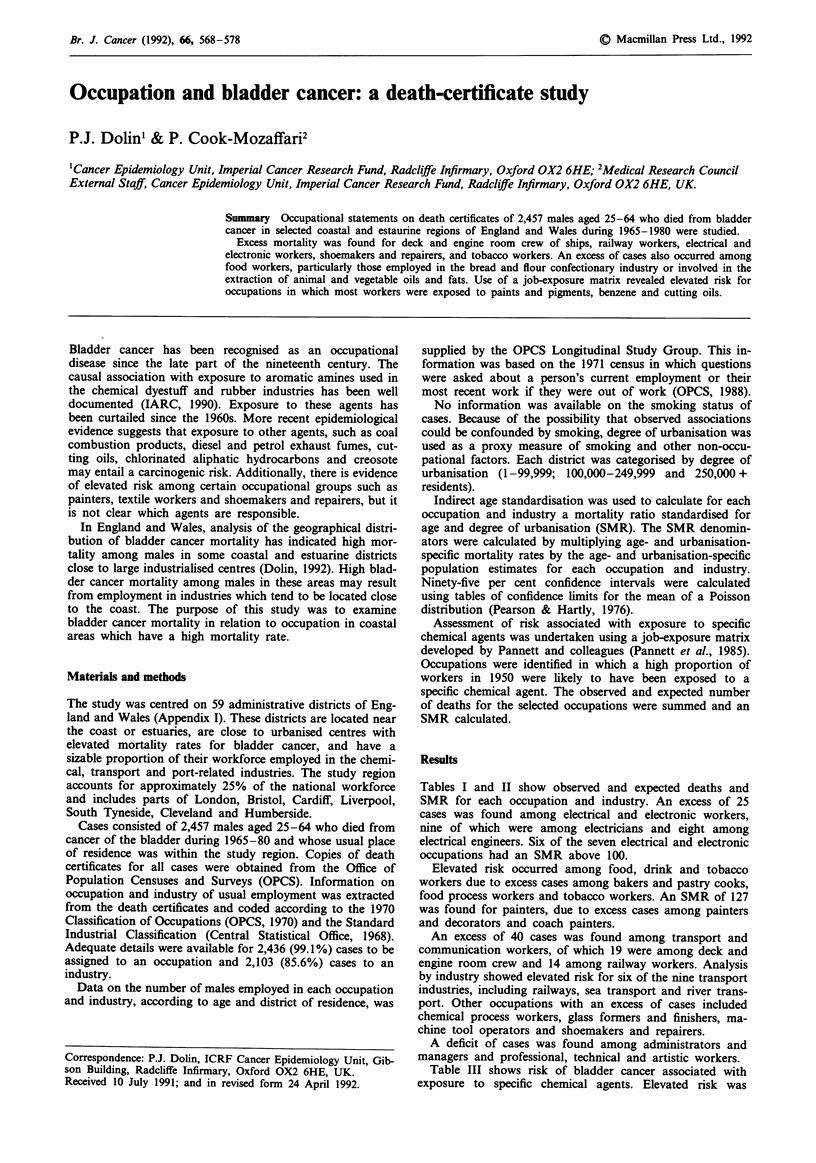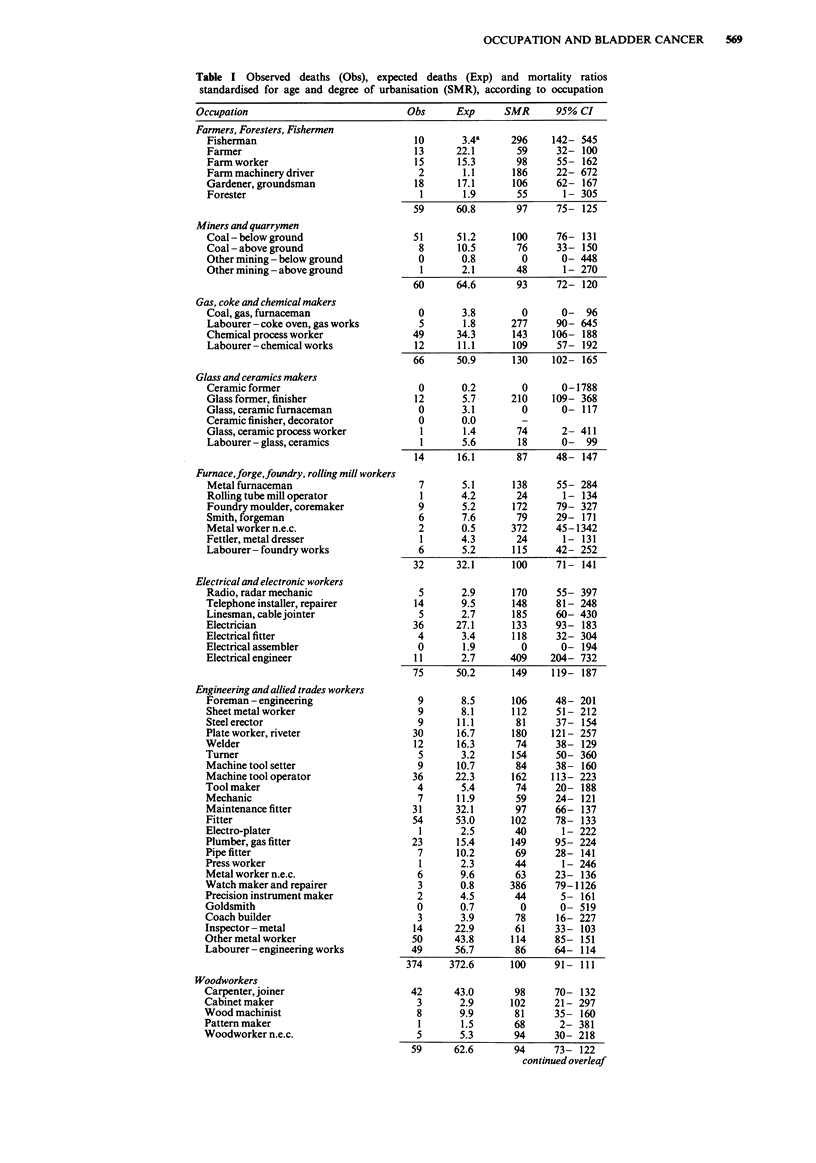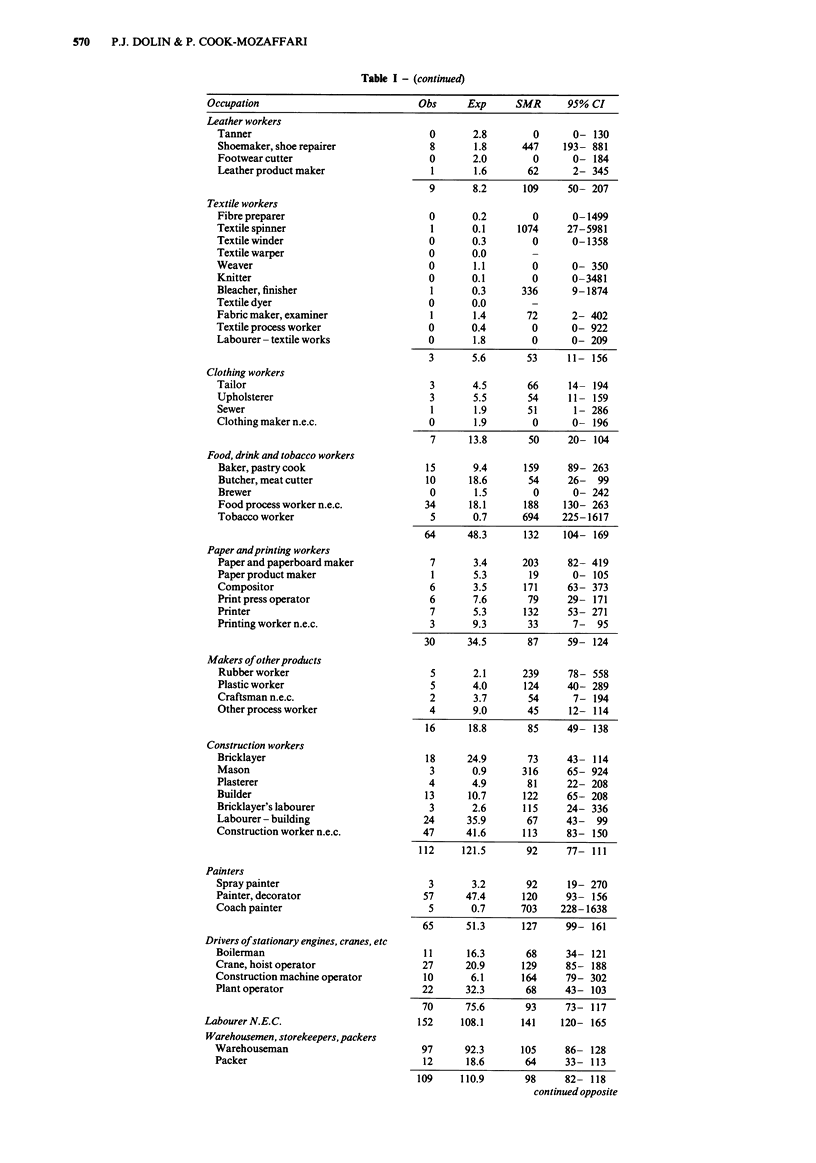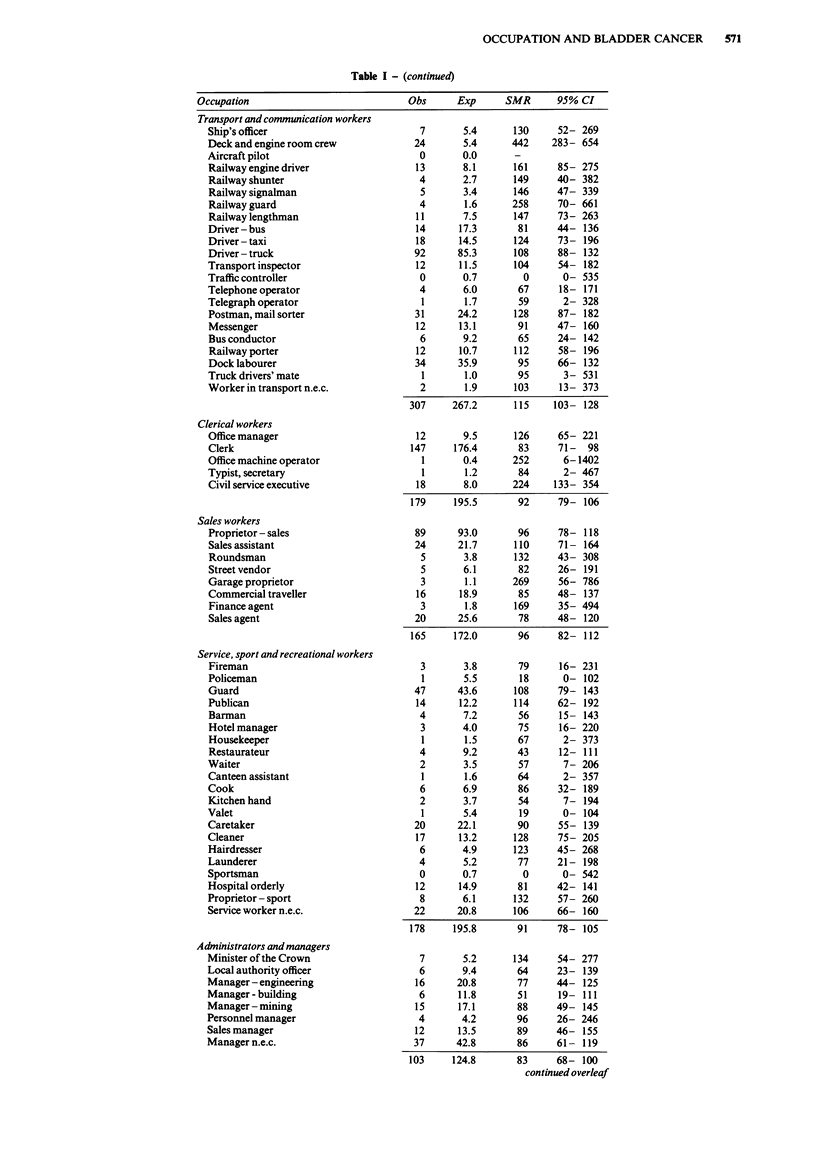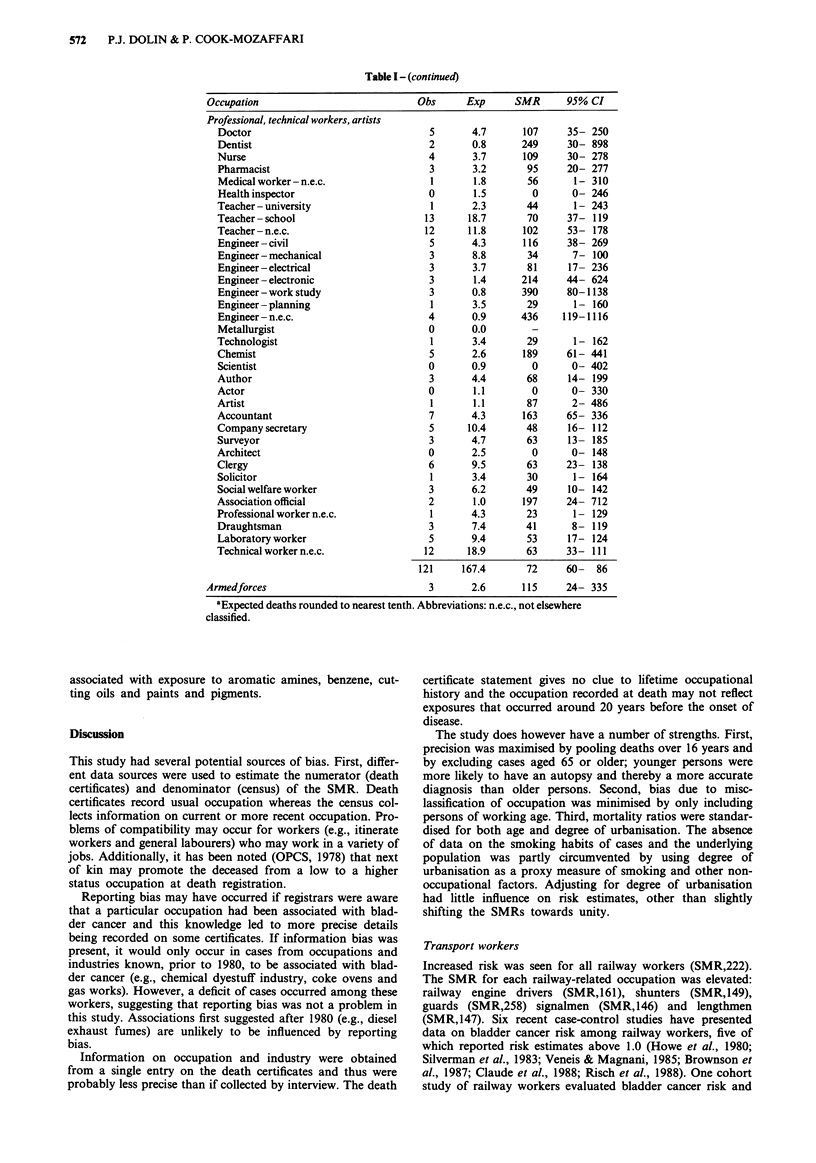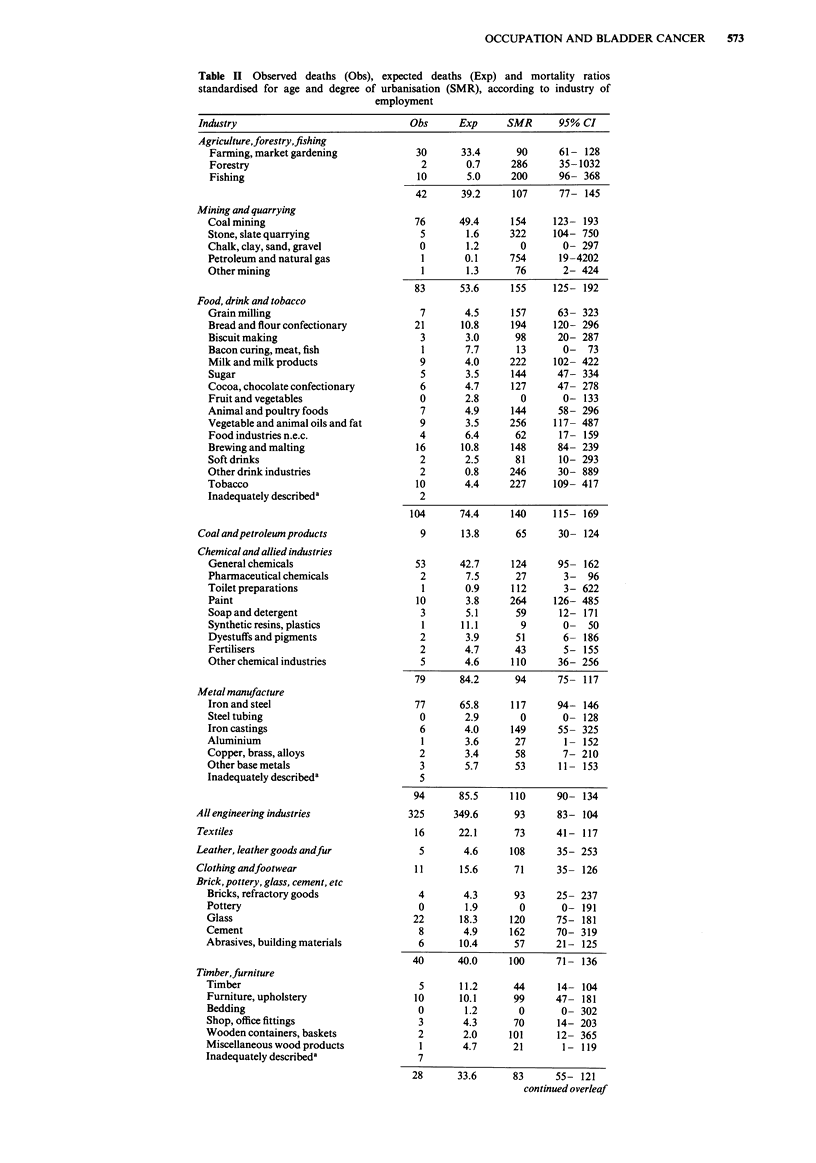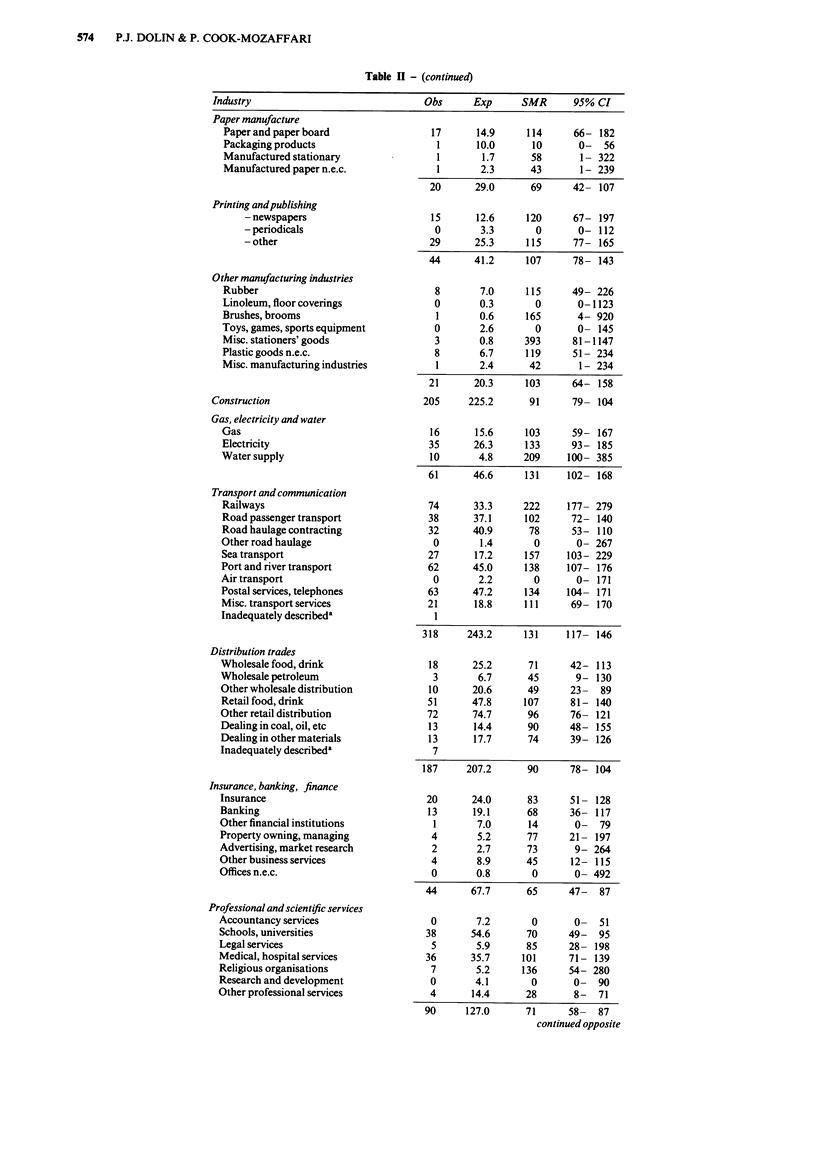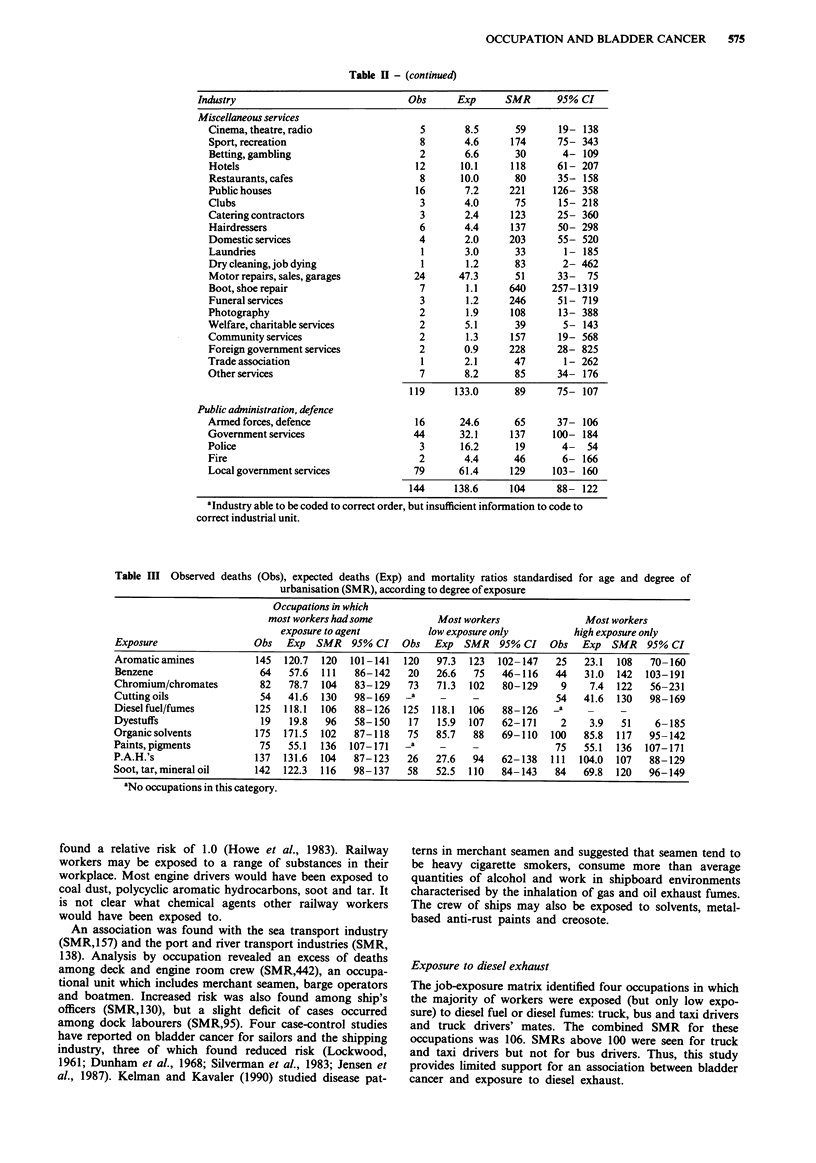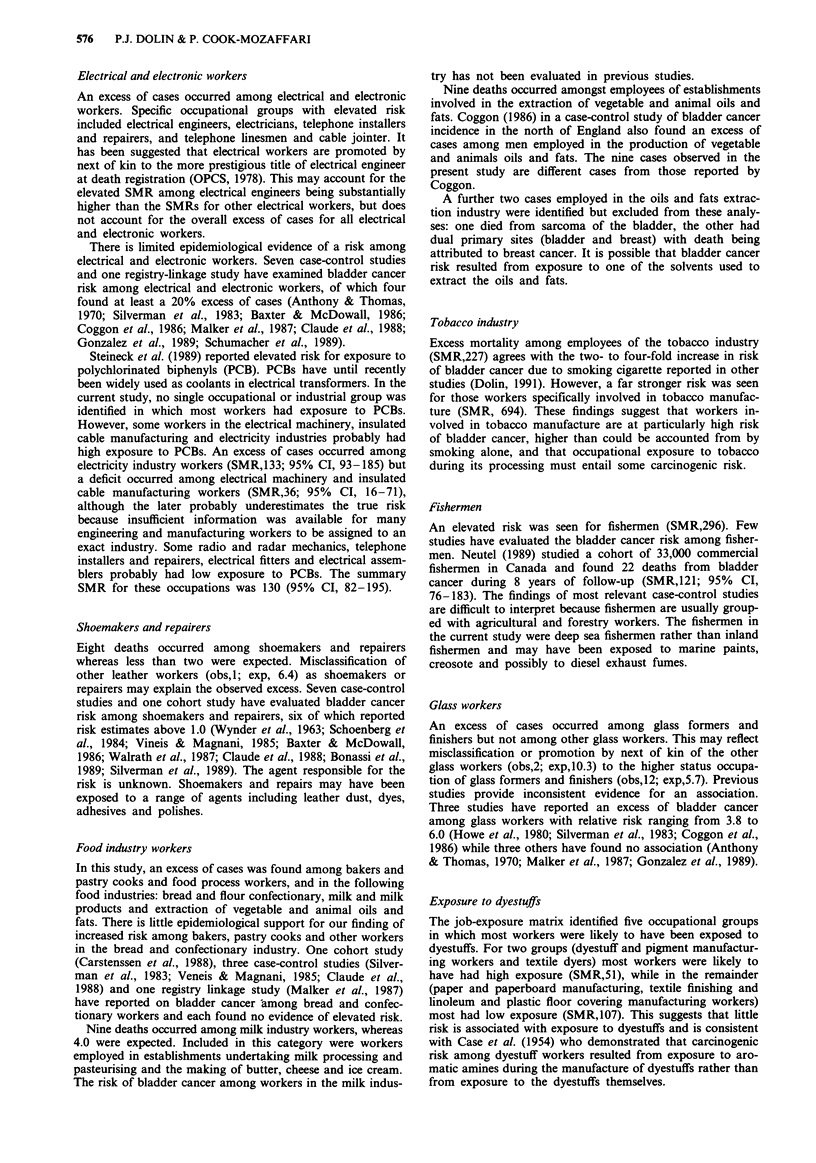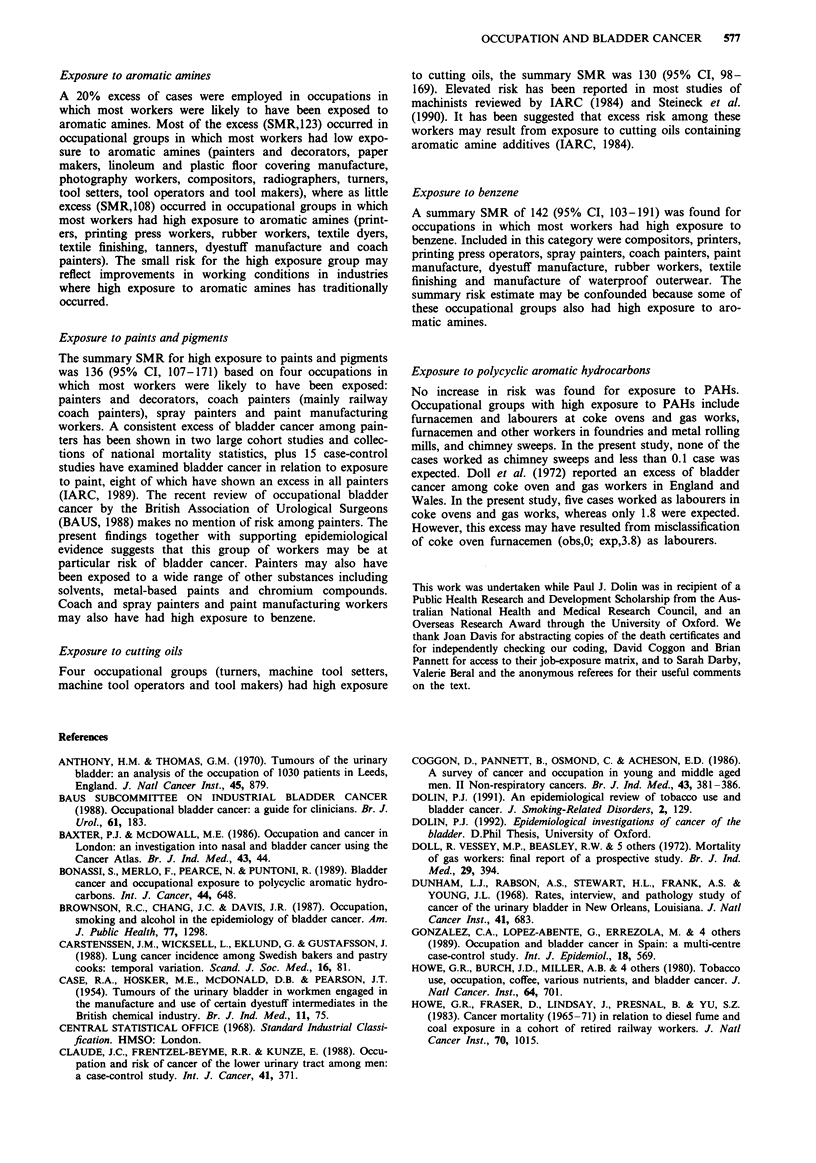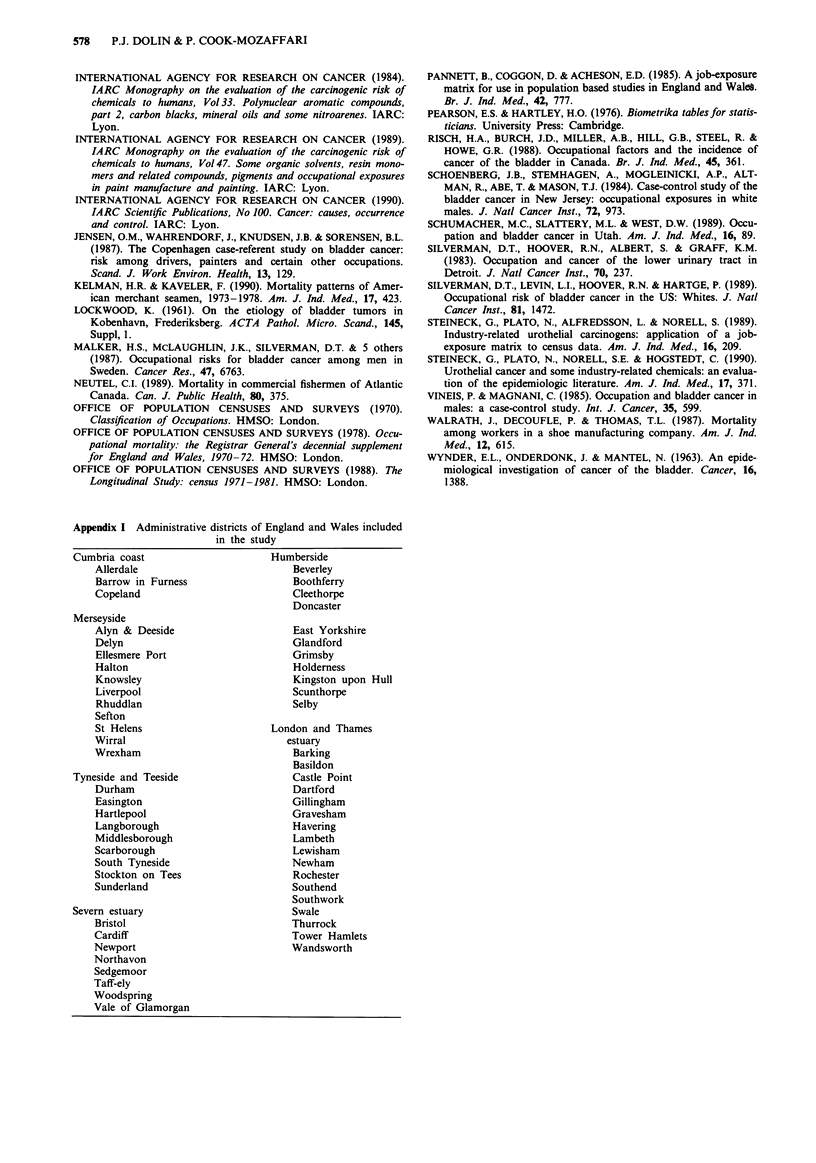# Occupation and bladder cancer: a death-certificate study.

**DOI:** 10.1038/bjc.1992.316

**Published:** 1992-09

**Authors:** P. J. Dolin, P. Cook-Mozaffari

**Affiliations:** Cancer Epidemiology Unit, Imperial Cancer Research Fund, Radcliffe Infirmary, Oxford, UK.

## Abstract

Occupational statements on death certificates of 2,457 males aged 25-64 who died from bladder cancer in selected coastal and estaurine regions of England and Wales during 1965-1980 were studied. Excess mortality was found for deck and engine room crew of ships, railway workers, electrical and electronic workers, shoemakers and repairers, and tobacco workers. An excess of cases also occurred among food workers, particularly those employed in the bread and flour confectionary industry or involved in the extraction of animal and vegetable oils and fats. Use of a job-exposure matrix revealed elevated risk for occupations in which most workers were exposed to paints and pigments, benzene and cutting oils.


Br. J. Cancer (1992), 66, 568-578 © Macmillan Press Ltd., 1992

### Occupation and bladder cancer: a death-certificate study

P.J. Dolin[1] & P. Cook-Mozaffari[2]

[1]Cancer Epidemiology Unit, Imperial Cancer Research Fund, Radcliffe Infirmary, Oxford OX2 6HE; [2]Medical Research Council External Staff, Cancer Epidemiology Unit, Imperial Cancer Research Fund, Radcliffe Infirmary, Oxford OX2 6HE, UK.

**Summary** Occupational statements on death certificates of 2,457 males aged 25-64 who died from bladder cancer in selected coastal and estaurine regions of England and Wales during 1965-1980 were studied.

Excess mortality was found for deck and engine room crew of ships, railway workers, electrical and electronic workers, shoemakers and repairers, and tobacco workers. An excess of cases also occurred among food workers, particularly those employed in the bread and flour confectionary industry or involved in the extraction of animal and vegetable oils and fats. Use of a job-exposure matrix revealed elevated risk for occupations in which most workers were exposed to paints and pigments, benzene and cutting oils.

Bladder cancer has been recognised as an occupational disease since the late part of the nineteenth century. The causal association with exposure to aromatic amines used in the chemical dyestuff and rubber industries has been well documented (IARC, 1990). Exposure to these agents has been curtailed since the 1960s. More recent epidemiological evidence suggests that exposure to other agents, such as coal combustion products, diesel and petrol exhaust fumes, cutting oils, chlorinated aliphatic hydrocarbons and creosote may entail a carcinogenic risk. Additionally, there is evidence of elevated risk among certain occupational groups such as painters, textile workers and shoemakers and repairers, but it is not clear which agents are responsible.

In England and Wales, analysis of the geographical distribution of bladder cancer mortality has indicated high mortality among males in some coastal and estuarine districts close to large industrialised centres (Dolin, 1992). High bladder cancer mortality among males in these areas may result from employment in industries which tend to be located close to the coast. The purpose of this study was to examine bladder cancer mortality in relation to occupation in coastal areas which have a high mortality rate.

### Materials and methods

The study was centred on 59 administrative districts of England and Wales (Appendix I). These districts are located near the coast or estuaries, are close to urbanised centres with elevated mortality rates for bladder cancer, and have a sizable proportion of their workforce employed in the chemical, transport and port-related industries. The study region accounts for approximately 25% of the national workforce and includes parts of London, Bristol, Cardiff, Liverpool, South Tyneside, Cleveland and Humberside.

Cases consisted of 2,457 males aged 25-64 who died from cancer of the bladder during 1965-80 and whose usual place of residence was within the study region. Copies of death certificates for all cases were obtained from the Office of Population Censuses and Surveys (OPCS). Information on occupation and industry of usual employment was extracted from the death certificates and coded according to the 1970 Classification of Occupations (OPCS, 1970) and the Standard Industrial Classification (Central Statistical Office, 1968). Adequate details were available for 2,436 (99.1%) cases to be assigned to an occupation and 2,103 (85.6%) cases to an industry.

Data on the number of males employed in each occupation and industry, according to age and district of residence, was supplied by the OPCS Longitudinal Study Group. This information was based on the 1971 census in which questions were asked about a person's current employment or their most recent work if they were out of work (OPCS, 1988).

No information was available on the smoking status of cases. Because of the possibility that observed associations could be confounded by smoking, degree of urbanisation was used as a proxy measure of smoking and other non-occupational factors. Each district was categorised by degree of urbanisation (1-99,999; 100,000-249,999 and 250,000+ residents).

Indirect age standardisation was used to calculate for each occupation and industry a mortality ratio standardised for age and degree of urbanisation (SMR). The SMR denominators were calculated by multiplying age- and urbanisation-specific mortality rates by the age- and urbanisation-specific population estimates for each occupation and industry. Ninety-five per cent confidence intervals were calculated using tables of confidence limits for the mean of a Poisson distribution (Pearson & Hartly, 1976).

Assessment of risk associated with exposure to specific chemical agents was undertaken using a job-exposure matrix developed by Pannett and colleagues (Pannett *et al.*, 1985). Occupations were identified in which a high proportion of workers in 1950 were likely to have been exposed to a specific chemical agent. The observed and expected number of deaths for the selected occupations were summed and an SMR calculated.

### Results

Tables I and II show observed and expected deaths and SMR for each occupation and industry. An excess of 25 cases was found among electrical and electronic workers, nine of which were among electricians and eight among electrical engineers. Six of the seven electrical and electronic occupations had an SMR above 100.

Elevated risk occurred among food, drink and tobacco workers due to excess cases among bakers and pastry cooks, food process workers and tobacco workers. An SMR of 127 was found for painters, due to excess cases among painters and decorators and coach painters.

An excess of 40 cases was found among transport and communication workers, of which 19 were among deck and engine room crew and 14 among railway workers. Analysis by industry showed elevated risk for six of the nine transport industries, including railways, sea transport and river transport. Other occupations with an excess of cases included chemical process workers, glass formers and finishers, machine tool operators and shoemakers and repairers.

A deficit of cases was found among administrators and managers and professional, technical and artistic workers.

Table III shows risk of bladder cancer associated with exposure to specific chemical agents. Elevated risk was

Correspondence: P.J. Dolin, ICRF Cancer Epidemiology Unit, Gibson Building, Radcliffe Infirmary, Oxford OX2 6HE, UK.
Received 10 July 1991; and in revised form 24 April 1992.

**Table I** Observed deaths (Obs), expected deaths (Exp) and mortality ratios standardised for age and degree of urbanisation (SMR), according to occupation

| *Occupation* | *Obs* | *Exp* | *SMR* | *95% CI* |
|---|---|---|---|---|
| *Farmers, Foresters, Fishermen* | | | | |
| Fisherman | 10 | 3.4[a] | 296 | 142– 545 |
| Farmer | 13 | 22.1 | 59 | 32– 100 |
| Farm worker | 15 | 15.3 | 98 | 55– 162 |
| Farm machinery driver | 2 | 1.1 | 186 | 22– 672 |
| Gardener, groundsman | 18 | 17.1 | 106 | 62– 167 |
| Forester | 1 | 1.9 | 55 | 1– 305 |
| | 59 | 60.8 | 97 | 75– 125 |
| *Miners and quarrymen* | | | | |
| Coal – below ground | 51 | 51.2 | 100 | 76– 131 |
| Coal – above ground | 8 | 10.5 | 76 | 33– 150 |
| Other mining – below ground | 0 | 0.8 | 0 | 0– 448 |
| Other mining – above ground | 1 | 2.1 | 48 | 1– 270 |
| | 60 | 64.6 | 93 | 72– 120 |
| *Gas, coke and chemical makers* | | | | |
| Coal, gas, furnaceman | 0 | 3.8 | 0 | 0– 96 |
| Labourer – coke oven, gas works | 5 | 1.8 | 277 | 90– 645 |
| Chemical process worker | 49 | 34.3 | 143 | 106– 188 |
| Labourer – chemical works | 12 | 11.1 | 109 | 57– 192 |
| | 66 | 50.9 | 130 | 102– 165 |
| *Glass and ceramics makers* | | | | |
| Ceramic former | 0 | 0.2 | 0 | 0–1788 |
| Glass former, finisher | 12 | 5.7 | 210 | 109– 368 |
| Glass, ceramic furnaceman | 0 | 3.1 | 0 | 0– 117 |
| Ceramic finisher, decorator | 0 | 0.0 | – | |
| Glass, ceramic process worker | 1 | 1.4 | 74 | 2– 411 |
| Labourer – glass, ceramics | 1 | 5.6 | 18 | 0– 99 |
| | 14 | 16.1 | 87 | 48– 147 |
| *Furnace, forge, foundry, rolling mill workers* | | | | |
| Metal furnaceman | 7 | 5.1 | 138 | 55– 284 |
| Rolling tube mill operator | 1 | 4.2 | 24 | 1– 134 |
| Foundry moulder, coremaker | 9 | 5.2 | 172 | 79– 327 |
| Smith, forgeman | 6 | 7.6 | 79 | 29– 171 |
| Metal worker n.e.c. | 2 | 0.5 | 372 | 45–1342 |
| Fettler, metal dresser | 1 | 4.3 | 24 | 1– 131 |
| Labourer – foundry works | 6 | 5.2 | 115 | 42– 252 |
| | 32 | 32.1 | 100 | 71– 141 |
| *Electrical and electronic workers* | | | | |
| Radio, radar mechanic | 5 | 2.9 | 170 | 55– 397 |
| Telephone installer, repairer | 14 | 9.5 | 148 | 81– 248 |
| Linesman, cable jointer | 5 | 2.7 | 185 | 60– 430 |
| Electrician | 36 | 27.1 | 133 | 93– 183 |
| Electrical fitter | 4 | 3.4 | 118 | 32– 304 |
| Electrical assembler | 0 | 1.9 | 0 | 0– 194 |
| Electrical engineer | 11 | 2.7 | 409 | 204– 732 |
| | 75 | 50.2 | 149 | 119– 187 |
| *Engineering and allied trades workers* | | | | |
| Foreman – engineering | 9 | 8.5 | 106 | 48– 201 |
| Sheet metal worker | 9 | 8.1 | 112 | 51– 212 |
| Steel erector | 9 | 11.1 | 81 | 37– 154 |
| Plate worker, riveter | 30 | 16.7 | 180 | 121– 257 |
| Welder | 12 | 16.3 | 74 | 38– 129 |
| Turner | 5 | 3.2 | 154 | 50– 360 |
| Machine tool setter | 9 | 10.7 | 84 | 38– 160 |
| Machine tool operator | 36 | 22.3 | 162 | 113– 223 |
| Tool maker | 4 | 5.4 | 74 | 20– 188 |
| Mechanic | 7 | 11.9 | 59 | 24– 121 |
| Maintenance fitter | 31 | 32.1 | 97 | 66– 137 |
| Fitter | 54 | 53.0 | 102 | 78– 133 |
| Electro-plater | 1 | 2.5 | 40 | 1– 222 |
| Plumber, gas fitter | 23 | 15.4 | 149 | 95– 224 |
| Pipe fitter | 7 | 10.2 | 69 | 28– 141 |
| Press worker | 1 | 2.3 | 44 | 1– 246 |
| Metal worker n.e.c. | 6 | 9.6 | 63 | 23– 136 |
| Watch maker and repairer | 3 | 0.8 | 386 | 79–1126 |
| Precision instrument maker | 2 | 4.5 | 44 | 5– 161 |
| Goldsmith | 0 | 0.7 | 0 | 0– 519 |
| Coach builder | 3 | 3.9 | 78 | 16– 227 |
| Inspector – metal | 14 | 22.9 | 61 | 33– 103 |
| Other metal worker | 50 | 43.8 | 114 | 85– 151 |
| Labourer – engineering works | 49 | 56.7 | 86 | 64– 114 |
| | 374 | 372.6 | 100 | 91– 111 |
| *Woodworkers* | | | | |
| Carpenter, joiner | 42 | 43.0 | 98 | 70– 132 |
| Cabinet maker | 3 | 2.9 | 102 | 21– 297 |
| Wood machinist | 8 | 9.9 | 81 | 35– 160 |
| Pattern maker | 1 | 1.5 | 68 | 2– 381 |
| Woodworker n.e.c. | 5 | 5.3 | 94 | 30– 218 |
| | 59 | 62.6 | 94 | 73– 122 |

*continued overleaf*

**Table I** – (*continued*)

| *Occupation* | *Obs* | *Exp* | *SMR* | *95% CI* |
|---|---|---|---|---|
| *Leather workers* | | | | |
| Tanner | 0 | 2.8 | 0 | 0– 130 |
| Shoemaker, shoe repairer | 8 | 1.8 | 447 | 193– 881 |
| Footwear cutter | 0 | 2.0 | 0 | 0– 184 |
| Leather product maker | 1 | 1.6 | 62 | 2– 345 |
| | 9 | 8.2 | 109 | 50– 207 |
| *Textile workers* | | | | |
| Fibre preparer | 0 | 0.2 | 0 | 0–1499 |
| Textile spinner | 1 | 0.1 | 1074 | 27–5981 |
| Textile winder | 0 | 0.3 | 0 | 0–1358 |
| Textile warper | 0 | 0.0 | – | |
| Weaver | 0 | 1.1 | 0 | 0– 350 |
| Knitter | 0 | 0.1 | 0 | 0–3481 |
| Bleacher, finisher | 1 | 0.3 | 336 | 9–1874 |
| Textile dyer | 0 | 0.0 | – | |
| Fabric maker, examiner | 1 | 1.4 | 72 | 2– 402 |
| Textile process worker | 0 | 0.4 | 0 | 0– 922 |
| Labourer – textile works | 0 | 1.8 | 0 | 0– 209 |
| | 3 | 5.6 | 53 | 11– 156 |
| *Clothing workers* | | | | |
| Tailor | 3 | 4.5 | 66 | 14– 194 |
| Upholsterer | 3 | 5.5 | 54 | 11– 159 |
| Sewer | 1 | 1.9 | 51 | 1– 286 |
| Clothing maker n.e.c. | 0 | 1.9 | 0 | 0– 196 |
| | 7 | 13.8 | 50 | 20– 104 |
| *Food, drink and tobacco workers* | | | | |
| Baker, pastry cook | 15 | 9.4 | 159 | 89– 263 |
| Butcher, meat cutter | 10 | 18.6 | 54 | 26– 99 |
| Brewer | 0 | 1.5 | 0 | 0– 242 |
| Food process worker n.e.c. | 34 | 18.1 | 188 | 130– 263 |
| Tobacco worker | 5 | 0.7 | 694 | 225–1617 |
| | 64 | 48.3 | 132 | 104– 169 |
| *Paper and printing workers* | | | | |
| Paper and paperboard maker | 7 | 3.4 | 203 | 82– 419 |
| Paper product maker | 1 | 5.3 | 19 | 0– 105 |
| Compositor | 6 | 3.5 | 171 | 63– 373 |
| Print press operator | 6 | 7.6 | 79 | 29– 171 |
| Printer | 7 | 5.3 | 132 | 53– 271 |
| Printing worker n.e.c. | 3 | 9.3 | 33 | 7– 95 |
| | 30 | 34.5 | 87 | 59– 124 |
| *Makers of other products* | | | | |
| Rubber worker | 5 | 2.1 | 239 | 78– 558 |
| Plastic worker | 5 | 4.0 | 124 | 40– 289 |
| Craftsman n.e.c. | 2 | 3.7 | 54 | 7– 194 |
| Other process worker | 4 | 9.0 | 45 | 12– 114 |
| | 16 | 18.8 | 85 | 49– 138 |
| *Construction workers* | | | | |
| Bricklayer | 18 | 24.9 | 73 | 43– 114 |
| Mason | 3 | 0.9 | 316 | 65– 924 |
| Plasterer | 4 | 4.9 | 81 | 22– 208 |
| Builder | 13 | 10.7 | 122 | 65– 208 |
| Bricklayer's labourer | 3 | 2.6 | 115 | 24– 336 |
| Labourer – building | 24 | 35.9 | 67 | 43– 99 |
| Construction worker n.e.c. | 47 | 41.6 | 113 | 83– 150 |
| | 112 | 121.5 | 92 | 77– 111 |
| *Painters* | | | | |
| Spray painter | 3 | 3.2 | 92 | 19– 270 |
| Painter, decorator | 57 | 47.4 | 120 | 93– 156 |
| Coach painter | 5 | 0.7 | 703 | 228–1638 |
| | 65 | 51.3 | 127 | 99– 161 |
| *Drivers of stationary engines, cranes, etc* | | | | |
| Boilerman | 11 | 16.3 | 68 | 34– 121 |
| Crane, hoist operator | 27 | 20.9 | 129 | 85– 188 |
| Construction machine operator | 10 | 6.1 | 164 | 79– 302 |
| Plant operator | 22 | 32.3 | 68 | 43– 103 |
| | 70 | 75.6 | 93 | 73– 117 |
| *Labourer N.E.C.* | 152 | 108.1 | 141 | 120– 165 |
| *Warehousemen, storekeepers, packers* | | | | |
| Warehouseman | 97 | 92.3 | 105 | 86– 128 |
| Packer | 12 | 18.6 | 64 | 33– 113 |
| | 109 | 110.9 | 98 | 82– 118 |

*continued opposite*

**Table I** – (*continued*)

| *Occupation* | *Obs* | *Exp* | *SMR* | *95% CI* |
|---|---|---|---|---|
| *Transport and communication workers* | | | | |
| Ship's officer | 7 | 5.4 | 130 | 52– 269 |
| Deck and engine room crew | 24 | 5.4 | 442 | 283– 654 |
| Aircraft pilot | 0 | 0.0 | – | |
| Railway engine driver | 13 | 8.1 | 161 | 85– 275 |
| Railway shunter | 4 | 2.7 | 149 | 40– 382 |
| Railway signalman | 5 | 3.4 | 146 | 47– 339 |
| Railway guard | 4 | 1.6 | 258 | 70– 661 |
| Railway lengthman | 11 | 7.5 | 147 | 73– 263 |
| Driver – bus | 14 | 17.3 | 81 | 44– 136 |
| Driver – taxi | 18 | 14.5 | 124 | 73– 196 |
| Driver – truck | 92 | 85.3 | 108 | 88– 132 |
| Transport inspector | 12 | 11.5 | 104 | 54– 182 |
| Traffic controller | 0 | 0.7 | 0 | 0– 535 |
| Telephone operator | 4 | 6.0 | 67 | 18– 171 |
| Telegraph operator | 1 | 1.7 | 59 | 2– 328 |
| Postman, mail sorter | 31 | 24.2 | 128 | 87– 182 |
| Messenger | 12 | 13.1 | 91 | 47– 160 |
| Bus conductor | 6 | 9.2 | 65 | 24– 142 |
| Railway porter | 12 | 10.7 | 112 | 58– 196 |
| Dock labourer | 34 | 35.9 | 95 | 66– 132 |
| Truck drivers' mate | 1 | 1.0 | 95 | 3– 531 |
| Worker in transport n.e.c. | 2 | 1.9 | 103 | 13– 373 |
| | 307 | 267.2 | 115 | 103– 128 |
| *Clerical workers* | | | | |
| Office manager | 12 | 9.5 | 126 | 65– 221 |
| Clerk | 147 | 176.4 | 83 | 71– 98 |
| Office machine operator | 1 | 0.4 | 252 | 6–1402 |
| Typist, secretary | 1 | 1.2 | 84 | 2– 467 |
| Civil service executive | 18 | 8.0 | 224 | 133– 354 |
| | 179 | 195.5 | 92 | 79– 106 |
| *Sales workers* | | | | |
| Proprietor – sales | 89 | 93.0 | 96 | 78– 118 |
| Sales assistant | 24 | 21.7 | 110 | 71– 164 |
| Roundsman | 5 | 3.8 | 132 | 43– 308 |
| Street vendor | 5 | 6.1 | 82 | 26– 191 |
| Garage proprietor | 3 | 1.1 | 269 | 56– 786 |
| Commercial traveller | 16 | 18.9 | 85 | 48– 137 |
| Finance agent | 3 | 1.8 | 169 | 35– 494 |
| Sales agent | 20 | 25.6 | 78 | 48– 120 |
| | 165 | 172.0 | 96 | 82– 112 |
| *Service, sport and recreational workers* | | | | |
| Fireman | 3 | 3.8 | 79 | 16– 231 |
| Policeman | 1 | 5.5 | 18 | 0– 102 |
| Guard | 47 | 43.6 | 108 | 79– 143 |
| Publican | 14 | 12.2 | 114 | 62– 192 |
| Barman | 4 | 7.2 | 56 | 15– 143 |
| Hotel manager | 3 | 4.0 | 75 | 16– 220 |
| Housekeeper | 1 | 1.5 | 67 | 2– 373 |
| Restaurateur | 4 | 9.2 | 43 | 12– 111 |
| Waiter | 2 | 3.5 | 57 | 7– 206 |
| Canteen assistant | 1 | 1.6 | 64 | 2– 357 |
| Cook | 6 | 6.9 | 86 | 32– 189 |
| Kitchen hand | 2 | 3.7 | 54 | 7– 194 |
| Valet | 1 | 5.4 | 19 | 0– 104 |
| Caretaker | 20 | 22.1 | 90 | 55– 139 |
| Cleaner | 17 | 13.2 | 128 | 75– 205 |
| Hairdresser | 6 | 4.9 | 123 | 45– 268 |
| Launderer | 4 | 5.2 | 77 | 21– 198 |
| Sportsman | 0 | 0.7 | 0 | 0– 542 |
| Hospital orderly | 12 | 14.9 | 81 | 42– 141 |
| Proprietor – sport | 8 | 6.1 | 132 | 57– 260 |
| Service worker n.e.c. | 22 | 20.8 | 106 | 66– 160 |
| | 178 | 195.8 | 91 | 78– 105 |
| *Administrators and managers* | | | | |
| Minister of the Crown | 7 | 5.2 | 134 | 54– 277 |
| Local authority officer | 6 | 9.4 | 64 | 23– 139 |
| Manager – engineering | 16 | 20.8 | 77 | 44– 125 |
| Manager - building | 6 | 11.8 | 51 | 19– 111 |
| Manager – mining | 15 | 17.1 | 88 | 49– 145 |
| Personnel manager | 4 | 4.2 | 96 | 26– 246 |
| Sales manager | 12 | 13.5 | 89 | 46– 155 |
| Manager n.e.c. | 37 | 42.8 | 86 | 61– 119 |
| | 103 | 124.8 | 83 | 68– 100 |

*continued overleaf*

**Table I** – (*continued*)

| *Occupation* | *Obs* | *Exp* | *SMR* | *95% CI* |
|---|---|---|---|---|
| *Professional, technical workers, artists* | | | | |
| Doctor | 5 | 4.7 | 107 | 35– 250 |
| Dentist | 2 | 0.8 | 249 | 30– 898 |
| Nurse | 4 | 3.7 | 109 | 30– 278 |
| Pharmacist | 3 | 3.2 | 95 | 20– 277 |
| Medical worker – n.e.c. | 1 | 1.8 | 56 | 1– 310 |
| Health inspector | 0 | 1.5 | 0 | 0– 246 |
| Teacher – university | 1 | 2.3 | 44 | 1– 243 |
| Teacher – school | 13 | 18.7 | 70 | 37– 119 |
| Teacher – n.e.c. | 12 | 11.8 | 102 | 53– 178 |
| Engineer – civil | 5 | 4.3 | 116 | 38– 269 |
| Engineer – mechanical | 3 | 8.8 | 34 | 7– 100 |
| Engineer – electrical | 3 | 3.7 | 81 | 17– 236 |
| Engineer – electronic | 3 | 1.4 | 214 | 44– 624 |
| Engineer – work study | 3 | 0.8 | 390 | 80–1138 |
| Engineer – planning | 1 | 3.5 | 29 | 1– 160 |
| Engineer – n.e.c. | 4 | 0.9 | 436 | 119–1116 |
| Metallurgist | 0 | 0.0 | – | |
| Technologist | 1 | 3.4 | 29 | 1– 162 |
| Chemist | 5 | 2.6 | 189 | 61– 441 |
| Scientist | 0 | 0.9 | 0 | 0– 402 |
| Author | 3 | 4.4 | 68 | 14– 199 |
| Actor | 0 | 1.1 | 0 | 0– 330 |
| Artist | 1 | 1.1 | 87 | 2– 486 |
| Accountant | 7 | 4.3 | 163 | 65– 336 |
| Company secretary | 5 | 10.4 | 48 | 16– 112 |
| Surveyor | 3 | 4.7 | 63 | 13– 185 |
| Architect | 0 | 2.5 | 0 | 0– 148 |
| Clergy | 6 | 9.5 | 63 | 23– 138 |
| Solicitor | 1 | 3.4 | 30 | 1– 164 |
| Social welfare worker | 3 | 6.2 | 49 | 10– 142 |
| Association official | 2 | 1.0 | 197 | 24– 712 |
| Professional worker n.e.c. | 1 | 4.3 | 23 | 1– 129 |
| Draughtsman | 3 | 7.4 | 41 | 8– 119 |
| Laboratory worker | 5 | 9.4 | 53 | 17– 124 |
| Technical worker n.e.c. | 12 | 18.9 | 63 | 33– 111 |
| | 121 | 167.4 | 72 | 60– 86 |
| *Armed forces* | 3 | 2.6 | 115 | 24– 335 |

[a]Expected deaths rounded to nearest tenth. Abbreviations: n.e.c., not elsewhere classified.

associated with exposure to aromatic amines, benzene, cutting oils and paints and pigments.

### Discussion

This study had several potential sources of bias. First, different data sources were used to estimate the numerator (death certificates) and denominator (census) of the SMR. Death certificates record usual occupation whereas the census collects information on current or more recent occupation. Problems of compatibility may occur for workers (e.g., itinerate workers and general labourers) who may work in a variety of jobs. Additionally, it has been noted (OPCS, 1978) that next of kin may promote the deceased from a low to a higher status occupation at death registration.

Reporting bias may have occurred if registrars were aware that a particular occupation had been associated with bladder cancer and this knowledge led to more precise details being recorded on some certificates. If information bias was present, it would only occur in cases from occupations and industries known, prior to 1980, to be associated with bladder cancer (e.g., chemical dyestuff industry, coke ovens and gas works). However, a deficit of cases occurred among these workers, suggesting that reporting bias was not a problem in this study. Associations first suggested after 1980 (e.g., diesel exhaust fumes) are unlikely to be influenced by reporting bias.

Information on occupation and industry were obtained from a single entry on the death certificates and thus were probably less precise than if collected by interview. The death certificate statement gives no clue to lifetime occupational history and the occupation recorded at death may not reflect exposures that occurred around 20 years before the onset of disease.

The study does however have a number of strengths. First, precision was maximised by pooling deaths over 16 years and by excluding cases aged 65 or older; younger persons were more likely to have an autopsy and thereby a more accurate diagnosis than older persons. Second, bias due to misclassification of occupation was minimised by only including persons of working age. Third, mortality ratios were standardised for both age and degree of urbanisation. The absence of data on the smoking habits of cases and the underlying population was partly circumvented by using degree of urbanisation as a proxy measure of smoking and other non-occupational factors. Adjusting for degree of urbanisation had little influence on risk estimates, other than slightly shifting the SMRs towards unity.

### *Transport workers*

Increased risk was seen for all railway workers (SMR,222). The SMR for each railway-related occupation was elevated: railway engine drivers (SMR,161), shunters (SMR,149), guards (SMR,258) signalmen (SMR,146) and lengthmen (SMR,147). Six recent case-control studies have presented data on bladder cancer risk among railway workers, five of which reported risk estimates above 1.0 (Howe *et al.*, 1980; Silverman *et al.*, 1983; Veneis & Magnani, 1985; Brownson *et al.*, 1987; Claude *et al.*, 1988; Risch *et al.*, 1988). One cohort study of railway workers evaluated bladder cancer risk and

**Table II** Observed deaths (Obs), expected deaths (Exp) and mortality ratios standardised for age and degree of urbanisation (SMR), according to industry of employment

| *Industry* | *Obs* | *Exp* | *SMR* | *95% CI* |
|---|---|---|---|---|
| *Agriculture, forestry, fishing* | | | | |
| Farming, market gardening | 30 | 33.4 | 90 | 61– 128 |
| Forestry | 2 | 0.7 | 286 | 35–1032 |
| Fishing | 10 | 5.0 | 200 | 96– 368 |
| | 42 | 39.2 | 107 | 77– 145 |
| *Mining and quarrying* | | | | |
| Coal mining | 76 | 49.4 | 154 | 123– 193 |
| Stone, slate quarrying | 5 | 1.6 | 322 | 104– 750 |
| Chalk, clay, sand, gravel | 0 | 1.2 | 0 | 0– 297 |
| Petroleum and natural gas | 1 | 0.1 | 754 | 19–4202 |
| Other mining | 1 | 1.3 | 76 | 2– 424 |
| | 83 | 53.6 | 155 | 125– 192 |
| *Food, drink and tobacco* | | | | |
| Grain milling | 7 | 4.5 | 157 | 63– 323 |
| Bread and flour confectionary | 21 | 10.8 | 194 | 120– 296 |
| Biscuit making | 3 | 3.0 | 98 | 20– 287 |
| Bacon curing, meat, fish | 1 | 7.7 | 13 | 0– 73 |
| Milk and milk products | 9 | 4.0 | 222 | 102– 422 |
| Sugar | 5 | 3.5 | 144 | 47– 334 |
| Cocoa, chocolate confectionary | 6 | 4.7 | 127 | 47– 278 |
| Fruit and vegetables | 0 | 2.8 | 0 | 0– 133 |
| Animal and poultry foods | 7 | 4.9 | 144 | 58– 296 |
| Vegetable and animal oils and fat | 9 | 3.5 | 256 | 117– 487 |
| Food industries n.e.c. | 4 | 6.4 | 62 | 17– 159 |
| Brewing and malting | 16 | 10.8 | 148 | 84– 239 |
| Soft drinks | 2 | 2.5 | 81 | 10– 293 |
| Other drink industries | 2 | 0.8 | 246 | 30– 889 |
| Tobacco | 10 | 4.4 | 227 | 109– 417 |
| Inadequately described[a] | 2 | | | |
| | 104 | 74.4 | 140 | 115– 169 |
| *Coal and petroleum products* | 9 | 13.8 | 65 | 30– 124 |
| *Chemical and allied industries* | | | | |
| General chemicals | 53 | 42.7 | 124 | 95– 162 |
| Pharmaceutical chemicals | 2 | 7.5 | 27 | 3– 96 |
| Toilet preparations | 1 | 0.9 | 112 | 3– 622 |
| Paint | 10 | 3.8 | 264 | 126– 485 |
| Soap and detergent | 3 | 5.1 | 59 | 12– 171 |
| Synthetic resins, plastics | 1 | 11.1 | 9 | 0– 50 |
| Dyestuffs and pigments | 2 | 3.9 | 51 | 6– 186 |
| Fertilisers | 2 | 4.7 | 43 | 5– 155 |
| Other chemical industries | 5 | 4.6 | 110 | 36– 256 |
| | 79 | 84.2 | 94 | 75– 117 |
| *Metal manufacture* | | | | |
| Iron and steel | 77 | 65.8 | 117 | 94– 146 |
| Steel tubing | 0 | 2.9 | 0 | 0– 128 |
| Iron castings | 6 | 4.0 | 149 | 55– 325 |
| Aluminium | 1 | 3.6 | 27 | 1– 152 |
| Copper, brass, alloys | 2 | 3.4 | 58 | 7– 210 |
| Other base metals | 3 | 5.7 | 53 | 11– 153 |
| Inadequately described[a] | 5 | | | |
| | 94 | 85.5 | 110 | 90– 134 |
| *All engineering industries* | 325 | 349.6 | 93 | 83– 104 |
| *Textiles* | 16 | 22.1 | 73 | 41– 117 |
| *Leather, leather goods and fur* | 5 | 4.6 | 108 | 35– 253 |
| *Clothing and footwear* | 11 | 15.6 | 71 | 35– 126 |
| *Brick, pottery, glass, cement, etc* | | | | |
| Bricks, refractory goods | 4 | 4.3 | 93 | 25– 237 |
| Pottery | 0 | 1.9 | 0 | 0– 191 |
| Glass | 22 | 18.3 | 120 | 75– 181 |
| Cement | 8 | 4.9 | 162 | 70– 319 |
| Abrasives, building materials | 6 | 10.4 | 57 | 21– 125 |
| | 40 | 40.0 | 100 | 71– 136 |
| *Timber, furniture* | | | | |
| Timber | 5 | 11.2 | 44 | 14– 104 |
| Furniture, upholstery | 10 | 10.1 | 99 | 47– 181 |
| Bedding | 0 | 1.2 | 0 | 0– 302 |
| Shop, office fittings | 3 | 4.3 | 70 | 14– 203 |
| Wooden containers, baskets | 2 | 2.0 | 101 | 12– 365 |
| Miscellaneous wood products | 1 | 4.7 | 21 | 1– 119 |
| Inadequately described[a] | 7 | | | |
| | 28 | 33.6 | 83 | 55– 121 |

*continued overleaf*

**Table II** – (*continued*)

| *Industry* | *Obs* | *Exp* | *SMR* | *95% CI* |
|---|---|---|---|---|
| *Paper manufacture* | | | | |
| Paper and paper board | 17 | 14.9 | 114 | 66– 182 |
| Packaging products | 1 | 10.0 | 10 | 0– 56 |
| Manufactured stationary | 1 | 1.7 | 58 | 1– 322 |
| Manufactured paper n.e.c. | 1 | 2.3 | 43 | 1– 239 |
| | 20 | 29.0 | 69 | 42– 107 |
| *Printing and publishing* | | | | |
| – newspapers | 15 | 12.6 | 120 | 67– 197 |
| – periodicals | 0 | 3.3 | 0 | 0– 112 |
| – other | 29 | 25.3 | 115 | 77– 165 |
| | 44 | 41.2 | 107 | 78– 143 |
| *Other manufacturing industries* | | | | |
| Rubber | 8 | 7.0 | 115 | 49– 226 |
| Linoleum, floor coverings | 0 | 0.3 | 0 | 0–1123 |
| Brushes, brooms | 1 | 0.6 | 165 | 4– 920 |
| Toys, games, sports equipment | 0 | 2.6 | 0 | 0– 145 |
| Misc. stationers' goods | 3 | 0.8 | 393 | 81–1147 |
| Plastic goods n.e.c. | 8 | 6.7 | 119 | 51– 234 |
| Misc. manufacturing industries | 1 | 2.4 | 42 | 1– 234 |
| | 21 | 20.3 | 103 | 64– 158 |
| *Construction* | 205 | 225.2 | 91 | 79– 104 |
| *Gas, electricity and water* | | | | |
| Gas | 16 | 15.6 | 103 | 59– 167 |
| Electricity | 35 | 26.3 | 133 | 93– 185 |
| Water supply | 10 | 4.8 | 209 | 100– 385 |
| | 61 | 46.6 | 131 | 102– 168 |
| *Transport and communication* | | | | |
| Railways | 74 | 33.3 | 222 | 177– 279 |
| Road passenger transport | 38 | 37.1 | 102 | 72– 140 |
| Road haulage contracting | 32 | 40.9 | 78 | 53– 110 |
| Other road haulage | 0 | 1.4 | 0 | 0– 267 |
| Sea transport | 27 | 17.2 | 157 | 103– 229 |
| Port and river transport | 62 | 45.0 | 138 | 107– 176 |
| Air transport | 0 | 2.2 | 0 | 0– 171 |
| Postal services, telephones | 63 | 47.2 | 134 | 104– 171 |
| Misc. transport services | 21 | 18.8 | 111 | 69– 170 |
| Inadequately described[a] | 1 | | | |
| | 318 | 243.2 | 131 | 117– 146 |
| *Distribution trades* | | | | |
| Wholesale food, drink | 18 | 25.2 | 71 | 42– 113 |
| Wholesale petroleum | 3 | 6.7 | 45 | 9– 130 |
| Other wholesale distribution | 10 | 20.6 | 49 | 23– 89 |
| Retail food, drink | 51 | 47.8 | 107 | 81– 140 |
| Other retail distribution | 72 | 74.7 | 96 | 76– 121 |
| Dealing in coal, oil, etc | 13 | 14.4 | 90 | 48– 155 |
| Dealing in other materials | 13 | 17.7 | 74 | 39– 126 |
| Inadequately described[a] | 7 | | | |
| | 187 | 207.2 | 90 | 78– 104 |
| *Insurance, banking, finance* | | | | |
| Insurance | 20 | 24.0 | 83 | 51– 128 |
| Banking | 13 | 19.1 | 68 | 36– 117 |
| Other financial institutions | 1 | 7.0 | 14 | 0– 79 |
| Property owning, managing | 4 | 5.2 | 77 | 21– 197 |
| Advertising, market research | 2 | 2.7 | 73 | 9– 264 |
| Other business services | 4 | 8.9 | 45 | 12– 115 |
| Offices n.e.c. | 0 | 0.8 | 0 | 0– 492 |
| | 44 | 67.7 | 65 | 47– 87 |
| *Professional and scientific services* | | | | |
| Accountancy services | 0 | 7.2 | 0 | 0– 51 |
| Schools, universities | 38 | 54.6 | 70 | 49– 95 |
| Legal services | 5 | 5.9 | 85 | 28– 198 |
| Medical, hospital services | 36 | 35.7 | 101 | 71– 139 |
| Religious organisations | 7 | 5.2 | 136 | 54– 280 |
| Research and development | 0 | 4.1 | 0 | 0– 90 |
| Other professional services | 4 | 14.4 | 28 | 8– 71 |
| | 90 | 127.0 | 71 | 58– 87 |

*continued opposite*

**Table II** – (*continued*)

| *Industry* | *Obs* | *Exp* | *SMR* | *95% CI* |
|---|---|---|---|---|
| *Miscellaneous services* | | | | |
| Cinema, theatre, radio | 5 | 8.5 | 59 | 19–138 |
| Sport, recreation | 8 | 4.6 | 174 | 75–343 |
| Betting, gambling | 2 | 6.6 | 30 | 4–109 |
| Hotels | 12 | 10.1 | 118 | 61–207 |
| Restaurants, cafes | 8 | 10.0 | 80 | 35–158 |
| Public houses | 16 | 7.2 | 221 | 126–358 |
| Clubs | 3 | 4.0 | 75 | 15–218 |
| Catering contractors | 3 | 2.4 | 123 | 25–360 |
| Hairdressers | 6 | 4.4 | 137 | 50–298 |
| Domestic services | 4 | 2.0 | 203 | 55–520 |
| Laundries | 1 | 3.0 | 33 | 1–185 |
| Dry cleaning, job dying | 1 | 1.2 | 83 | 2–462 |
| Motor repairs, sales, garages | 24 | 47.3 | 51 | 33–75 |
| Boot, shoe repair | 7 | 1.1 | 640 | 257–1319 |
| Funeral services | 3 | 1.2 | 246 | 51–719 |
| Photography | 2 | 1.9 | 108 | 13–388 |
| Welfare, charitable services | 2 | 5.1 | 39 | 5–143 |
| Community services | 2 | 1.3 | 157 | 19–568 |
| Foreign government services | 2 | 0.9 | 228 | 28–825 |
| Trade association | 1 | 2.1 | 47 | 1–262 |
| Other services | 7 | 8.2 | 85 | 34–176 |
| | 119 | 133.0 | 89 | 75–107 |
| *Public administration, defence* | | | | |
| Armed forces, defence | 16 | 24.6 | 65 | 37–106 |
| Government services | 44 | 32.1 | 137 | 100–184 |
| Police | 3 | 16.2 | 19 | 4–54 |
| Fire | 2 | 4.4 | 46 | 6–166 |
| Local government services | 79 | 61.4 | 129 | 103–160 |
| | 144 | 138.6 | 104 | 88–122 |

[a]Industry able to be coded to correct order, but insufficient information to code to correct industrial unit.

**Table III** Observed deaths (Obs), expected deaths (Exp) and mortality ratios standardised for age and degree of urbanisation (SMR), according to degree of exposure

| | *Occupations in which most workers had some exposure to agent* | | | | *Most workers low exposure only* | | | | *Most workers high exposure only* | | | |
|---|---|---|---|---|---|---|---|---|---|---|---|---|
| *Exposure* | *Obs* | *Exp* | *SMR* | *95% CI* | *Obs* | *Exp* | *SMR* | *95% CI* | *Obs* | *Exp* | *SMR* | *95% CI* |
| Aromatic amines | 145 | 120.7 | 120 | 101–141 | 120 | 97.3 | 123 | 102–147 | 25 | 23.1 | 108 | 70–160 |
| Benzene | 64 | 57.6 | 111 | 86–142 | 20 | 26.6 | 75 | 46–116 | 44 | 31.0 | 142 | 103–191 |
| Chromium/chromates | 82 | 78.7 | 104 | 83–129 | 73 | 71.3 | 102 | 80–129 | 9 | 7.4 | 122 | 56–231 |
| Cutting oils | 54 | 41.6 | 130 | 98–169 | –[a] | – | – | | 54 | 41.6 | 130 | 98–169 |
| Diesel fuel/fumes | 125 | 118.1 | 106 | 88–126 | 125 | 118.1 | 106 | 88–126 | –[a] | – | – | |
| Dyestuffs | 19 | 19.8 | 96 | 58–150 | 17 | 15.9 | 107 | 62–171 | 2 | 3.9 | 51 | 6–185 |
| Organic solvents | 175 | 171.5 | 102 | 87–118 | 75 | 85.7 | 88 | 69–110 | 100 | 85.8 | 117 | 95–142 |
| Paints, pigments | 75 | 55.1 | 136 | 107–171 | –[a] | – | – | | 75 | 55.1 | 136 | 107–171 |
| P.A.H.'s | 137 | 131.6 | 104 | 87–123 | 26 | 27.6 | 94 | 62–138 | 111 | 104.0 | 107 | 88–129 |
| Soot, tar, mineral oil | 142 | 122.3 | 116 | 98–137 | 58 | 52.5 | 110 | 84–143 | 84 | 69.8 | 120 | 96–149 |

[a]No occupations in this category.

found a relative risk of 1.0 (Howe *et al.*, 1983). Railway workers may be exposed to a range of substances in their workplace. Most engine drivers would have been exposed to coal dust, polycyclic aromatic hydrocarbons, soot and tar. It is not clear what chemical agents other railway workers would have been exposed to.

An association was found with the sea transport industry (SMR,157) and the port and river transport industries (SMR, 138). Analysis by occupation revealed an excess of deaths among deck and engine room crew (SMR,442), an occupational unit which includes merchant seamen, barge operators and boatmen. Increased risk was also found among ship's officers (SMR,130), but a slight deficit of cases occurred among dock labourers (SMR,95). Four case-control studies have reported on bladder cancer for sailors and the shipping industry, three of which found reduced risk (Lockwood, 1961; Dunham *et al.*, 1968; Silverman *et al.*, 1983; Jensen *et al.*, 1987). Kelman and Kavaler (1990) studied disease patterns in merchant seamen and suggested that seamen tend to be heavy cigarette smokers, consume more than average quantities of alcohol and work in shipboard environments characterised by the inhalation of gas and oil exhaust fumes. The crew of ships may also be exposed to solvents, metal-based anti-rust paints and creosote.

### *Exposure to diesel exhaust*

The job-exposure matrix identified four occupations in which the majority of workers were exposed (but only low exposure) to diesel fuel or diesel fumes: truck, bus and taxi drivers and truck drivers' mates. The combined SMR for these occupations was 106. SMRs above 100 were seen for truck and taxi drivers but not for bus drivers. Thus, this study provides limited support for an association between bladder cancer and exposure to diesel exhaust.

*Electrical and electronic workers*

An excess of cases occurred among electrical and electronic workers. Specific occupational groups with elevated risk included electrical engineers, electricians, telephone installers and repairers, and telephone linesmen and cable jointer. It has been suggested that electrical workers are promoted by next of kin to the more prestigious title of electrical engineer at death registration (OPCS, 1978). This may account for the elevated SMR among electrical engineers being substantially higher than the SMRs for other electrical workers, but does not account for the overall excess of cases for all electrical and electronic workers.

There is limited epidemiological evidence of a risk among electrical and electronic workers. Seven case-control studies and one registry-linkage study have examined bladder cancer risk among electrical and electronic workers, of which four found at least a 20% excess of cases (Anthony & Thomas, 1970; Silverman *et al.*, 1983; Baxter & McDowall, 1986; Coggon *et al.*, 1986; Malker *et al.*, 1987; Claude *et al.*, 1988; Gonzalez *et al.*, 1989; Schumacher *et al.*, 1989).

Steineck *et al.* (1989) reported elevated risk for exposure to polychlorinated biphenyls (PCB). PCBs have until recently been widely used as coolants in electrical transformers. In the current study, no single occupational or industrial group was identified in which most workers had exposure to PCBs. However, some workers in the electrical machinery, insulated cable manufacturing and electricity industries probably had high exposure to PCBs. An excess of cases occurred among electricity industry workers (SMR,133; 95% CI, 93–185) but a deficit occurred among electrical machinery and insulated cable manufacturing workers (SMR,36; 95% CI, 16–71), although the later probably underestimates the true risk because insufficient information was available for many engineering and manufacturing workers to be assigned to an exact industry. Some radio and radar mechanics, telephone installers and repairers, electrical fitters and electrical assemblers probably had low exposure to PCBs. The summary SMR for these occupations was 130 (95% CI, 82–195).

*Shoemakers and repairers*

Eight deaths occurred among shoemakers and repairers whereas less than two were expected. Misclassification of other leather workers (obs,1; exp, 6.4) as shoemakers or repairers may explain the observed excess. Seven case-control studies and one cohort study have evaluated bladder cancer risk among shoemakers and repairers, six of which reported risk estimates above 1.0 (Wynder *et al.*, 1963; Schoenberg *et al.*, 1984; Vineis & Magnani, 1985; Baxter & McDowall, 1986; Walrath *et al.*, 1987; Claude *et al.*, 1988; Bonassi *et al.*, 1989; Silverman *et al.*, 1989). The agent responsible for the risk is unknown. Shoemakers and repairs may have been exposed to a range of agents including leather dust, dyes, adhesives and polishes.

*Food industry workers*

In this study, an excess of cases was found among bakers and pastry cooks and food process workers, and in the following food industries: bread and flour confectionary, milk and milk products and extraction of vegetable and animal oils and fats. There is little epidemiological support for our finding of increased risk among bakers, pastry cooks and other workers in the bread and confectionary industry. One cohort study (Carstenssen *et al.*, 1988), three case-control studies (Silverman *et al.*, 1983; Veneis & Magnani, 1985; Claude *et al.*, 1988) and one registry linkage study (Malker *et al.*, 1987) have reported on bladder cancer among bread and confectionary workers and each found no evidence of elevated risk.

Nine deaths occurred among milk industry workers, whereas 4.0 were expected. Included in this category were workers employed in establishments undertaking milk processing and pasteurising and the making of butter, cheese and ice cream. The risk of bladder cancer among workers in the milk industry has not been evaluated in previous studies.

Nine deaths occurred amongst employees of establishments involved in the extraction of vegetable and animal oils and fats. Coggon (1986) in a case-control study of bladder cancer incidence in the north of England also found an excess of cases among men employed in the production of vegetable and animals oils and fats. The nine cases observed in the present study are different cases from those reported by Coggon.

A further two cases employed in the oils and fats extraction industry were identified but excluded from these analyses: one died from sarcoma of the bladder, the other had dual primary sites (bladder and breast) with death being attributed to breast cancer. It is possible that bladder cancer risk resulted from exposure to one of the solvents used to extract the oils and fats.

*Tobacco industry*

Excess mortality among employees of the tobacco industry (SMR,227) agrees with the two- to four-fold increase in risk of bladder cancer due to smoking cigarette reported in other studies (Dolin, 1991). However, a far stronger risk was seen for those workers specifically involved in tobacco manufacture (SMR, 694). These findings suggest that workers involved in tobacco manufacture are at particularly high risk of bladder cancer, higher than could be accounted from by smoking alone, and that occupational exposure to tobacco during its processing must entail some carcinogenic risk.

*Fishermen*

An elevated risk was seen for fishermen (SMR,296). Few studies have evaluated the bladder cancer risk among fishermen. Neutel (1989) studied a cohort of 33,000 commercial fishermen in Canada and found 22 deaths from bladder cancer during 8 years of follow-up (SMR,121; 95% CI, 76–183). The findings of most relevant case-control studies are difficult to interpret because fishermen are usually grouped with agricultural and forestry workers. The fishermen in the current study were deep sea fishermen rather than inland fishermen and may have been exposed to marine paints, creosote and possibly to diesel exhaust fumes.

*Glass workers*

An excess of cases occurred among glass formers and finishers but not among other glass workers. This may reflect misclassification or promotion by next of kin of the other glass workers (obs,2; exp,10.3) to the higher status occupation of glass formers and finishers (obs,12; exp,5.7). Previous studies provide inconsistent evidence for an association. Three studies have reported an excess of bladder cancer among glass workers with relative risk ranging from 3.8 to 6.0 (Howe *et al.*, 1980; Silverman *et al.*, 1983; Coggon *et al.*, 1986) while three others have found no association (Anthony & Thomas, 1970; Malker *et al.*, 1987; Gonzalez *et al.*, 1989).

*Exposure to dyestuffs*

The job-exposure matrix identified five occupational groups in which most workers were likely to have been exposed to dyestuffs. For two groups (dyestuff and pigment manufacturing workers and textile dyers) most workers were likely to have had high exposure (SMR,51), while in the remainder (paper and paperboard manufacturing, textile finishing and linoleum and plastic floor covering manufacturing workers) most had low exposure (SMR,107). This suggests that little risk is associated with exposure to dyestuffs and is consistent with Case *et al.* (1954) who demonstrated that carcinogenic risk among dyestuff workers resulted from exposure to aromatic amines during the manufacture of dyestuffs rather than from exposure to the dyestuffs themselves.

*Exposure to aromatic amines*

A 20% excess of cases were employed in occupations in which most workers were likely to have been exposed to aromatic amines. Most of the excess (SMR,123) occurred in occupational groups in which most workers had low exposure to aromatic amines (painters and decorators, paper makers, linoleum and plastic floor covering manufacture, photography workers, compositors, radiographers, turners, tool setters, tool operators and tool makers), where as little excess (SMR,108) occurred in occupational groups in which most workers had high exposure to aromatic amines (printers, printing press workers, rubber workers, textile dyers, textile finishing, tanners, dyestuff manufacture and coach painters). The small risk for the high exposure group may reflect improvements in working conditions in industries where high exposure to aromatic amines has traditionally occurred.

*Exposure to paints and pigments*

The summary SMR for high exposure to paints and pigments was 136 (95% CI, 107–171) based on four occupations in which most workers were likely to have been exposed: painters and decorators, coach painters (mainly railway coach painters), spray painters and paint manufacturing workers. A consistent excess of bladder cancer among painters has been shown in two large cohort studies and collections of national mortality statistics, plus 15 case-control studies have examined bladder cancer in relation to exposure to paint, eight of which have shown an excess in all painters (IARC, 1989). The recent review of occupational bladder cancer by the British Association of Urological Surgeons (BAUS, 1988) makes no mention of risk among painters. The present findings together with supporting epidemiological evidence suggests that this group of workers may be at particular risk of bladder cancer. Painters may also have been exposed to a wide range of other substances including solvents, metal-based paints and chromium compounds. Coach and spray painters and paint manufacturing workers may also have had high exposure to benzene.

*Exposure to cutting oils*

Four occupational groups (turners, machine tool setters, machine tool operators and tool makers) had high exposure to cutting oils, the summary SMR was 130 (95% CI, 98–169). Elevated risk has been reported in most studies of machinists reviewed by IARC (1984) and Steineck *et al.* (1990). It has been suggested that excess risk among these workers may result from exposure to cutting oils containing aromatic amine additives (IARC, 1984).

*Exposure to benzene*

A summary SMR of 142 (95% CI, 103–191) was found for occupations in which most workers had high exposure to benzene. Included in this category were compositors, printers, printing press operators, spray painters, coach painters, paint manufacture, dyestuff manufacture, rubber workers, textile finishing and manufacture of waterproof outerwear. The summary risk estimate may be confounded because some of these occupational groups also had high exposure to aromatic amines.

*Exposure to polycyclic aromatic hydrocarbons*

No increase in risk was found for exposure to PAHs. Occupational groups with high exposure to PAHs include furnacemen and labourers at coke ovens and gas works, furnacemen and other workers in foundries and metal rolling mills, and chimney sweeps. In the present study, none of the cases worked as chimney sweeps and less than 0.1 case was expected. Doll *et al.* (1972) reported an excess of bladder cancer among coke oven and gas workers in England and Wales. In the present study, five cases worked as labourers in coke ovens and gas works, whereas only 1.8 were expected. However, this excess may have resulted from misclassification of coke oven furnacemen (obs,0; exp,3.8) as labourers.

This work was undertaken while Paul J. Dolin was in recipient of a Public Health Research and Development Scholarship from the Australian National Health and Medical Research Council, and an Overseas Research Award through the University of Oxford. We thank Joan Davis for abstracting copies of the death certificates and for independently checking our coding, David Coggon and Brian Pannett for access to their job-exposure matrix, and to Sarah Darby, Valerie Beral and the anonymous referees for their useful comments on the text.

**References**

ANTHONY, H.M. & THOMAS, G.M. (1970). Tumours of the urinary bladder: an analysis of the occupation of 1030 patients in Leeds, England. *J. Natl Cancer Inst.*, **45**, 879.

BAUS SUBCOMMITTEE ON INDUSTRIAL BLADDER CANCER (1988). Occupational bladder cancer: a guide for clinicians. *Br. J. Urol.*, **61**, 183.

BAXTER, P.J. & McDOWALL, M.E. (1986). Occupation and cancer in London: an investigation into nasal and bladder cancer using the Cancer Atlas. *Br. J. Ind. Med.*, **43**, 44.

BONASSI, S., MERLO, F., PEARCE, N. & PUNTONI, R. (1989). Bladder cancer and occupational exposure to polycyclic aromatic hydrocarbons. *Int. J. Cancer*, **44**, 648.

BROWNSON, R.C., CHANG, J.C. & DAVIS, J.R. (1987). Occupation, smoking and alcohol in the epidemiology of bladder cancer. *Am. J. Public Health*, **77**, 1298.

CARSTENSSEN, J.M., WICKSELL, L., EKLUND, G. & GUSTAFSSON, J. (1988). Lung cancer incidence among Swedish bakers and pastry cooks: temporal variation. *Scand. J. Soc. Med.*, **16**, 81.

CASE, R.A., HOSKER, M.E., McDONALD, D.B. & PEARSON, J.T. (1954). Tumours of the urinary bladder in workmen engaged in the manufacture and use of certain dyestuff intermediates in the British chemical industry. *Br. J. Ind. Med.*, **11**, 75.

CENTRAL STATISTICAL OFFICE (1968). *Standard Industrial Classification*. HMSO: London.

CLAUDE, J.C., FRENTZEL-BEYME, R.R. & KUNZE, E. (1988). Occupation and risk of cancer of the lower urinary tract among men: a case-control study. *Int. J. Cancer*, **41**, 371.

COGGON, D., PANNETT, B., OSMOND, C. & ACHESON, E.D. (1986). A survey of cancer and occupation in young and middle aged men. II Non-respiratory cancers. *Br. J. Ind. Med.*, **43**, 381–386.

DOLIN, P.J. (1991). An epidemiological review of tobacco use and bladder cancer. *J. Smoking-Related Disorders*, **2**, 129.

DOLIN, P.J. (1992). *Epidemiological investigations of cancer of the bladder*. D.Phil Thesis, University of Oxford.

DOLL, R. VESSEY, M.P., BEASLEY, R.W. & 5 others (1972). Mortality of gas workers: final report of a prospective study. *Br. J. Ind. Med.*, **29**, 394.

DUNHAM, L.J., RABSON, A.S., STEWART, H.L., FRANK, A.S. & YOUNG, J.L. (1968). Rates, interview, and pathology study of cancer of the urinary bladder in New Orleans, Louisiana. *J. Natl Cancer Inst.*, **41**, 683.

GONZALEZ, C.A., LOPEZ-ABENTE, G., ERREZOLA, M. & 4 others (1989). Occupation and bladder cancer in Spain: a multi-centre case-control study. *Int. J. Epidemiol.*, **18**, 569.

HOWE, G.R., BURCH, J.D., MILLER, A.B. & 4 others (1980). Tobacco use, occupation, coffee, various nutrients, and bladder cancer. *J. Natl Cancer. Inst.*, **64**, 701.

HOWE, G.R., FRASER, D., LINDSAY, J., PRESNAL, B. & YU, S.Z. (1983). Cancer mortality (1965–71) in relation to diesel fume and coal exposure in a cohort of retired railway workers. *J. Natl Cancer Inst.*, **70**, 1015.

INTERNATIONAL AGENCY FOR RESEARCH ON CANCER (1984). *IARC Monography on the evaluation of the carcinogenic risk of chemicals to humans, Vol 33. Polynuclear aromatic compounds, part 2, carbon blacks, mineral oils and some nitroarenes.* IARC: Lyon.

INTERNATIONAL AGENCY FOR RESEARCH ON CANCER (1989). *IARC Monography on the evaluation of the carcinogenic risk of chemicals to humans, Vol 47. Some organic solvents, resin monomers and related compounds, pigments and occupational exposures in paint manufacture and painting.* IARC: Lyon.

INTERNATIONAL AGENCY FOR RESEARCH ON CANCER (1990). *IARC Scientific Publications, No 100. Cancer: causes, occurrence and control.* IARC: Lyon.

JENSEN, O.M., WAHRENDORF, J., KNUDSEN, J.B. & SORENSEN, B.L. (1987). The Copenhagen case-referent study on bladder cancer: risk among drivers, painters and certain other occupations. *Scand. J. Work Environ. Health*, **13**, 129.

KELMAN, H.R. & KAVELER, F. (1990). Mortality patterns of American merchant seamen, 1973–1978. *Am. J. Ind. Med.*, **17**, 423.

LOCKWOOD, K. (1961). On the etiology of bladder tumors in Kobenhavn, Frederiksberg. *ACTA Pathol. Micro. Scand.*, **145**, Suppl, 1.

MALKER, H.S., MCLAUGHLIN, J.K., SILVERMAN, D.T. & 5 others (1987). Occupational risks for bladder cancer among men in Sweden. *Cancer Res.*, **47**, 6763.

NEUTEL, C.I. (1989). Mortality in commercial fishermen of Atlantic Canada. *Can. J. Public Health*, **80**, 375.

OFFICE OF POPULATION CENSUSES AND SURVEYS (1970). *Classification of Occupations.* HMSO: London.

OFFICE OF POPULATION CENSUSES AND SURVEYS (1978). *Occupational mortality: the Registrar General's decennial supplement for England and Wales, 1970–72.* HMSO: London.

OFFICE OF POPULATION CENSUSES AND SURVEYS (1988). *The Longitudinal Study: census 1971–1981.* HMSO: London.

PANNETT, B., COGGON, D. & ACHESON, E.D. (1985). A job-exposure matrix for use in population based studies in England and Wales. *Br. J. Ind. Med.*, **42**, 777.

PEARSON, E.S. & HARTLEY, H.O. (1976). *Biometrika tables for statisticians.* University Press: Cambridge.

RISCH, H.A., BURCH, J.D., MILLER, A.B., HILL, G.B., STEEL, R. & HOWE, G.R. (1988). Occupational factors and the incidence of cancer of the bladder in Canada. *Br. J. Ind. Med.*, **45**, 361.

SCHOENBERG, J.B., STEMHAGEN, A., MOGLEINICKI, A.P., ALTMAN, R., ABE, T. & MASON, T.J. (1984). Case-control study of the bladder cancer in New Jersey: occupational exposures in white males. *J. Natl Cancer Inst.*, **72**, 973.

SCHUMACHER, M.C., SLATTERY, M.L. & WEST, D.W. (1989). Occupation and bladder cancer in Utah. *Am. J. Ind. Med.*, **16**, 89.

SILVERMAN, D.T., HOOVER, R.N., ALBERT, S. & GRAFF, K.M. (1983). Occupation and cancer of the lower urinary tract in Detroit. *J. Natl Cancer Inst.*, **70**, 237.

SILVERMAN, D.T., LEVIN, L.I., HOOVER, R.N. & HARTGE, P. (1989). Occupational risk of bladder cancer in the US: Whites. *J. Natl Cancer Inst.*, **81**, 1472.

STEINECK, G., PLATO, N., ALFREDSSON, L. & NORELL, S. (1989). Industry-related urothelial carcinogens: application of a job-exposure matrix to census data. *Am. J. Ind. Med.*, **16**, 209.

STEINECK, G., PLATO, N., NORELL, S.E. & HOGSTEDT, C. (1990). Urothelial cancer and some industry-related chemicals: an evaluation of the epidemiologic literature. *Am. J. Ind. Med.*, **17**, 371.

VINEIS, P. & MAGNANI, C. (1985). Occupation and bladder cancer in males: a case-control study. *Int. J. Cancer*, **35**, 599.

WALRATH, J., DECOUFLE, P. & THOMAS, T.L. (1987). Mortality among workers in a shoe manufacturing company. *Am. J. Ind. Med.*, **12**, 615.

WYNDER, E.L., ONDERDONK, J. & MANTEL, N. (1963). An epidemiological investigation of cancer of the bladder. *Cancer*, **16**, 1388.

**Appendix I** Administrative districts of England and Wales included in the study

Cumbria coast

- Allerdale

- Barrow in Furness

- Copeland

Merseyside

- Alyn & Deeside

- Delyn

- Ellesmere Port

- Halton

- Knowsley

- Liverpool

- Rhuddlan

- Sefton

- St Helens